# cMyc increases cell number through uncoupling of cell division from cell size in CHO cells

**DOI:** 10.1186/1472-6750-9-76

**Published:** 2009-09-07

**Authors:** Darrin Kuystermans, Mohamed Al-Rubeai

**Affiliations:** 1 School of Chemical and Bioprocess Engineering, University College Dublin, Delfield, Dublin 4, Ireland; 2 Conway Institute of Biomolecular and Biomedical Research, University College Dublin, Belfield, Dublin 4, Ireland

## Abstract

**Background:**

Over the past decades, the increase in maximal cell numbers for the production of mammalian derived biologics has been in a large part due to the development of optimal feeding strategies. Engineering of the cell line is one of probable approaches for increasing cell numbers in bioreactor.

**Results:**

We have demonstrated that the over-expression of the *c-myc *gene in immortalised CHO cells can increase proliferation rate and maximal cell density in batch culture compared to the control. The changes were attributed to a rapid transition into S-phase from a shortened duration of G_1 _phase and to the uncoupling of cell size from cell proliferation. To achieve the >70% increase in maximal cell density without additional supply of nutrients the cells underwent an overall reduction of 14% in size as well as a significant decrease in glucose and amino acid consumption rate. Consequently, the total biomass accumulation did not show a significant change from the control. The amount of hSEAP-hFc activity of the over expressing *c-myc *cell line was found to be within 0.7% of the control.

**Conclusion:**

It is shown that the manipulation of cell cycle kinetics and indirectly cell metabolism gives higher cell densities in CHO batch cultures. The unaltered apoptotic rate supported the proposition that the increase in cell number was a result of enhance cell cycle kinetics and cellular metabolism rather than increasing viability. Production of hSEAP-hFc from a constitutive *c-myc *over-expressing cell line did not increase with the increase in cell number.

## Background

The Chinese Hamster Ovary (CHO) cell line is the workhorse of the bioprocessing industry for the production of biopharmaceuticals. The high productivity of the cell line in bioreactors can be said to be dependent on having a high viable cell density of rapidly dividing cells over an extended period of time. The relatively high cell numbers achieved over more than two decades of process optimization strategies utilising media and fed batch cultivation improvements [1,2] resulted in product titres of more than 5 g/l.

In biopharmaceutical production systems, optimisation of cell culture processes is an evolving field as the more we understand of the complex dynamics involved in regulating cell survival, growth and productivity, the better we can apply this knowledge to improve titre. Cell engineering strategies to improve maximal cell number would be a major step towards reaching the goal of increased product titre. The use of c-Myc has been recently put forward as a possible means of achieving this goal [3,4], but a more thorough understanding is needed to explain the role of *c-myc *in the CHO-K1 immortalised continuous cell line. Particularly, as a key regulator of the cell cycle and to provide a rationale for the experimental observation that the cell size seems to play an important role in establishing an increased cell number, before it can be incorporated into cell lines that are used for the production of therapeutic protein at industrial scale.

The product of *c-myc *is a nuclear phosphoprotein that has been implicated in cell cycle progression and apoptosis, although it is still unclear on how the protein carries out its functions and is regulated [5]. It belongs to a family of *myc *genes which was discovered as a cellular homologue to the viral oncogene (*v-myc*) of the avian myelocytomatosis MC29 retrovirus [6]. The nuclear phosphoprotein has a short life of about 20-30 minutes [7]. Its physiological role is based on the fact that its expression strongly correlates with cellular proliferation and thus it has been broadly expressed during embryogenesis and in tissue compartments that contain high proliferative capacity such as the mammalian gut and skin epidermis.

As a transcription factor, c-Myc binds to the promoters of a large number of genes to regulate their expression, in turn, controlling, the cell cycle, metabolic processes, macromolecular synthesis and apoptosis[8]. In quiescent G_0 _cells, *c-myc *expression is almost undetectable until there is some serum or mitogenic stimulation, which causes rapid induction of cell transition into G_1_. After this transition, c-Myc levels decline to a low but detectable level, while removal of serum or mitogenic stimuli will again put c-Myc into undetectable level and thus cellular arrest occurs. Some known targets of c-Myc are cyclin D2 and CDK2 (essential in cell cycle regulation), and eIF4 and eIF2 which are important in cell growth[9].

In the past few years it has become apparent that *c-myc *regulation is related somewhat to apoptotic pathways. Pro-apoptotic pathways have been identified which converges on the central mitochondrial induced apoptotic pathway. This is due to the fact that *c-myc *over-expression also affects the ARF/Mdm-2 pathway (ARF, a small protein from the INK4 family, binds to Mdm2 protein whose function is to negatively regulate the activity of p53 protein) by promoting expression of ARF and thus up-regulating p53 expression leading to apoptosis [10].

In immortalised continuous cell lines, such as CHO cell line, net expansion of a clone is not only achieved by an increased rate of proliferation but a decrease in the apoptotic rate as well. The most widely held opinion on this is that since the cell cycle and apoptotic pathways are coupled through several sub-networked pathways, the induction of the cell cycle can sensitize the cell to apoptosis but the apoptotic pathway is suppressed as long as the appropriate survival factors are available to the cell [8]. Earlier findings have established a role for *c-myc *in apoptosis in diverse settings through separate 'death priming' and 'death triggering' mechanisms in which 'death priming' and mitogenic signals are coordinated [11].

The objective of this study is to provide data for further understanding CHO cells with an increased proliferation capacity. This work will focus on the cell cycle and metabolic aspects of having a cell line reach higher viable cell densities with increased proliferation under the same supplemented media conditions. Since the variability of clones would not give further information related to this objective, the original transfected population is used. For the investigation into productivity clonal selection was used to ensure a stable producer of hSEAP fusion protein. Over-expression of *c-myc *has resulted in a reduced duration of the G_1 _phase of cell cycle thus leading to a reduced growth rate and increased maximum cell number. This change was associated with reduced glucose and amino acid consumption rate and decreased cell size. The results showed that the total volumetric biomass was negligibly affected by *c-myc *over-expression and that without nutrient addition any increase in cell number would be compensated by a comparable decrease in cell volume.

## Methods

All chemicals in the Materials and Methods section were obtained from Sigma Aldrich unless stated otherwise.

### Cell Transfection and Maintenance

The cMycCHO cell line resulted from calcium-mediated stable transfection of CHO-K1 with the DORclaG123 (c-Myc plasmid). The plasmid was kindly donated by Dr. T. Littlewood (Imperial Cancer Research Fund, UK). The surviving cells were pooled separately for each transfection and were maintained in Ham's F12 supplemented with 5% FCS. Using dilution cloning a stable cMycCHO clone was selected and used in subsequent experiments. A detailed transfection and selection procedure is described in a previous publication [3]. Selection pressure was maintained by incubation in DMEM/F12, 4 mM L-glutamine, 5% FBS (Lonza Biologics) and 1 mg/mL geneticin G418 every 10^th ^generation and cell samples were taken before and after incubation to ensure stable overexpression of the c-Myc protein. Selection pressure was removed 2 passages before an experimental run to negate the effects of the presence of antibiotic.

### Static Batch Culture and Cell Size of CHO-K1 and cMycCHO

CHO-K1 and cMycCHO were maintained in DMEM/F12 with 5% FBS (Lonza Biologics, UK) and 4 mM L-glutamine at 37°C vented at 5% CO_2_. Static cultures from each cell line were plated in 6 well plates, 80,000 cells/cm^2^, with an initial density of 2 × 10^5 ^cells/ml allowing triplicate cultures to be sacrificed at regular intervals for each cell line. A duplicate culture was plated from the experimental seeding inoculum for mid-exponential phase harvesting to carry out the western analysis. When harvesting cells, floating cells were collected by centrifugation of the aspirated medium, and adherent cells were detached by Accutase™ and collected by centrifugation, these were pooled together in a media volume equal to the original culture volume before cell counts. Viable cell concentration and viability were monitored using the trypan blue exclusion method [12]. All cell volume measurements were done using the coulter principle of electronic volume on a Quanta SC (Beckman Coulter) flow cytometer which has forward scatter replaced by electronic volume.

### Static Spent Media Cultures

The spent media was obtained from each respective culture to replicate conditions of supplement depletion. Each cell line was seeded in T-25 flasks with 10 ml of the spent media at 5 × 10^5 ^cells/ml at 37°C vented at 5% CO_2_. Cells were derived from a mid-exponential phase culture. Samples were taken at 0, 24, 44, and 118 hours for analysis of apoptosis.

### Transfection with the hSEAP-hFc gene

The plasmid, pFUSE-SEAP-hFc1, (InvivoGen, USA) was used to transfect cMycCHO and CHO-K1 cell lines using FuGENE^® ^6 transfection reagent (Roche, UK). Transfected cells were incubated in fresh DMEM/F12 (Sigma-Aldrich) with 5% heat inactivated FBS (PAA, UK) and 4 mM L-glutamine or 4 mM L-alanine-L-glutamine (Ultra-Glutamine, Lonza, UK) at a temperature of 37°C vented at 5% CO2. The culture medium was then replaced with fresh medium containing 400 ug/ml Zeocin™ and incubated at 37°C. After 3 days the transfected cells were selected in fresh medium containing 400 ug/ml Zeocin™ and 1 mg/ml G418 followed by isolating the population of cells by limiting dilution. Selection of stable clones was done by assessing for the highest SEAP activity for each cell line over a period of 6 weeks. To ensure that expression remained stable the cultures were cultured in the presence of G418 and Zeocin™ every 10^th ^generation.

### Western Analysis

Total cellular protein was prepared from cells washed in 0.35 M sucrose and resuspended in 400 μl of lysis buffer (Ultrapure reagents from Amersham Biosciences, Piscataway, NJ): 2% (w/v) CHAPS, 9.5 M urea, 0.8% (w/v) pharmalyte pH 3-10, 1% (w/v) DTT (Amersham Biosciences, UK) and 1 protease inhibitor tablet per 10 ml (Roche Molecular, Mannheim, Germany) for 30 minutes before subsequent 4°C centrifugation at 17,000 g for 30 minutes. Sample was stored at -80°C for minimum of 24 hours before concentration determination using a modified Bradford assay [13]. 50 μg/well underwent electrophoresis on a 12% sodium dodecyl sulfate-polyacrylamide (SDS-PAGE) mini gel (Pierce, Rockford, IL) after dilution in 1× Laemli buffer prior to denaturation at 95°C for 10 minutes. The gel was stained with Instantblue™ (Novexin Ltd, UK) for visual verification of loading before de-staining for electrotransfer. The protein was then transferred to nitrocellulose membrane (Amersham Biosciences, Little Chalfont, UK). Then, the membrane was incubated with the primary antibody, anti-myc 9E10 antibody (Santa Cruz Biotechnology, Santa Cruz, CA), and then incubated with a HRP conjugated anti-mouse whole IgG from goat as the secondary antibody. Bands were developed by chemiluminescent detection (SuperSignal West Pico System, Pierce) according to the manufacturer's instructions. The membrane was stained with Ponceau S to re-verify loading and uniformity of the electrotransfer.

### Static Cell Cycle Data

Flow cytometry was used to determine the percentage of cells in various phases of the cell cycle. Every 24 hours both CHO-K1 and cMycCHO were dissociated from the wells using Accutase™, centrifuged at 160 g for 5 minutes and were resuspended in 500 μl of Nuclear Isolation Media (NIM)-DAPI staining solution (NIM-DAPI, NPE Systems, Pembroke Pines, FL, USA) at a density of 1 × 10^6 ^cells/ml for 1 minute at room temperature. The suspension was then filtered through a 25 μm Filter Tip (NPE Systems). NIM-DAPI stained samples were run on a Beckman Coulter Quanta SC flow Cytometer fitted with a mercury arc lamp, a 357/22 nm exciter and a 465/30 nm emission filter. The G_1 _peak was fixed at a specific channel number and a minimum of 15,000 nuclear signals was collected. The distribution of cells in the G_1_, S, and G_2_/M phases was analyzed with the Multicycle software program (Phoenix Flow Systems, San Diego, CA).

### Analysis of Apoptosis

CHO-K1 and cMycCHO cells removed at different times during the batch cultures and cells incubated in spent media were analysed for the percentage of viable, apoptotic and necrotic cells. Dual staining for both Annexin-V binding to phosphatidylserine (PS) surface proteins by Annexin-V- Fluorescein isothiocyanate (FITC) (BD Biosciences, UK) and cellular DNA using Propidium Iodide (PI) was performed as follows (Ishaque and Al-Rubeai, 1999): 1 × 10^6 ^cells/ml were washed in ice cold PBS (0.01 M phosphate buffer, 0.0027 M potassium chloride and 0.137 M sodium chloride, pH 7.4, at 25°C) and centrifuged at 300 g for 5 minutes at 4°C for resuspension in 1× binding buffer ((10 mM HEPES solution) pH 7.4, 140 mM NaCl, and 2.5 mM CaCl_2_). 1 μl of Annexin-V FITC was added to 100 μl of cell suspension and incubated for 10 minutes at 20-25°C before adding 400 ul of 1× binding buffer and 10 μl of PI (50 ug/ml) which was incubated for 1 minute before analysis. The sample was analysed with a Beckman flow cytometer equipped with a 488 nm diode laser. A 525 band pass detection filter was used to obtain FITC fluorescence and a 670 nm long pass filter is used to collect the maximal emission of PI fluorescence [14]. Fluorescence compensation was carried out before taking final readings.

### Cell Cycle Kinetics Labelling

A BrdU (5-Bromo-2'-deoxyuridine) pulse labelling and staining procedure for kinetic analysis of the cell cycle was initiated the 2nd day after initial seeding of the cultures in flasks. 20 μM of BrdU (BD Biosciences, UK) was added to the exponentially growing cells. After a labelling pulse of 1 hour at 37°C, cells were washed with fresh medium and then incubated in the corresponding medium for 1, 3, and 5 hours with samples taken from triplicate wells. After the indicated BrdU-free chase time, cells were harvested and washed twice in ice-cold PBS and centrifuged at 500 g for 5 minutes. The cells were then resuspended in 500 μl ice-cold PBS and 5 ml 70% ethanol was added drop-wise before leaving the suspension on ice for 15 minutes. The samples were stored in a -20°C for 1 week after fixation. The staining procedure to determine the DNA and BrdU content was a modified procedure from Istfan N.W et al. [15]. Briefly, the fixed samples were centrifuged for 10 minutes at 500 g and then washed twice with cold PBS. To this 3 ml of 2N HCl was added and left at room temperature in the dark on a shaker for 30 minutes before washing with PBS and then adding 3 ml of 0.1 M Borax to neutralize the acid at room temperature for 2-4 minutes. 3 ml of 0.5% Triton X-100 was added and left for 10 minutes at room temperature on a shaker after a PBS wash. Afterwards, the pellet was resuspended in 0.1 ml of PBS and 20 μl of anti-BrdU FITC (BD Bioscience) was added directly before incubation for 30 minutes at room temperature in the dark. The sample was then washed with PBS and the pellet resuspended in 0.5-1 ml of PBS before adding 50 μl of RNase A solution per tube and incubated for 30 minutes at 37°C. From a 1 mg/ml stock, 25 μl of PI stain solution was added per tube and left on ice for 30 minutes before bivariate DNA-BrdU analysis was carried out on the flow cytometer.

### Cell Cycle Kinetic Analysis

Dual stained samples were analysed with a Beckman flow cytometer equipped with a 488 nm diode laser, A 525 band pass detection filter was used to obtain green fluorescence from FITC-labeled anti-BrdU and a 670 nm long pass filter is used to collect the maximal emission of PI red fluorescence. The red fluorescence was calibrated by setting the G_1 _peak to a fixed channel number and green fluorescence was calibrated using an unlabeled control. The cell size was also taken simultaneously at 48 and 53 hours (exponential phase) with these samples to determine the relative cell size in each phase. Cell Lab Quanta SC software (Beckman Coulter, UK) and the computer program WinMDI V2.8 (The Scripps Research Institute) was used to analysis the cytometric histograms. Calculations was carried out using the equations previously described [16-18]. Briefly, the bivariate BrdU-DNA contour plot obtained with WinMDI V2.8 had the cells separated on the x-axis according to DNA content and the y-axis according to BrdU incorporation. Gating was established for G_1_, G_2_/M, labeled undivided (f_lu_) cells, and labeled divided (f_ld_) cells. First, the relative movement (RM) at each pulse time point of the cells during the S-phase to G1 and G2 was calculated using the following equation:

(1)

Where F_L_(t) is the mean red fluorescence at time (t) while F_G1 _and F_G2+M _are the mean red fluorescence of cells in each respective phase. This can be related to the following linear relationship:

(2)

Second, the function v [16] a quantity (= cT_S_) represented by the following equations is obtained:

(3a)

&

(3b)

Equation 3a is used when the times are less than the time for the G_2+M _phase (T_G2+M_) and equation 3b otherwise[16]. Here the fraction of labeled undivided (f^lu^), labeled undivided in G_2_+M phase (f^lu^_G2+M_), and labeled divided (f^ld^) are obtained from the bivariate BrdU-DNA contour plot.

The potential cell cycle time (T_pot_)[15] can be calculated using the value for v and TS in the following equation:

(4)

To determine the calculated time for the cell spent in G2 and M phase an analytical procedure described in White et al, 2000 was used. This relies on the calculation of the solution to a cubic equation where the cell production rate (c) is obtained through a relation between RM(t), v, and f^lu^[18]:

(5)

Where

(6)

After determining the cell production rate the following equation is used to determine T_G2+M_:

(7)

After determining T_G2+M _and T_S _the time spent in G_1 _could be calculated by subtraction of both calculated times from T_pot_.

### Determination of Extracellular Glucose, Lactate, Ammonia and Amino Acid Concentrations

Glucose was measured with an Ascencia contour (Bayer Diagnostics, Ireland) glucose meter with the provided pre-calibrated Microfill test strips (Bayer Diagnostics, Ireland). Lactate was measured using the Accutrend Lactate Meter together with BM-Lactate Strips (Roche Diagnostics GmbH, Germany). Briefly, The BM-Lactate calibration strip was used to calibrate the instrument to the accompanied BM-Lactate strips. Lactate was determined by reflectance photometry at a wave length of 657 nm via a colorimetric-oxidase mediator reaction. L-Glutamine and ammonia were measured with a Megazyme kit K-ASNAM (Megazyme, Ireland) using supernatant from each day of the culture. The amino acids samples were taken at the end of exponential phase by centrifuging 1.5 ml of culture for 5 minutes at 180 g and storing the supernatant at -20°C for 1 week before analyses. Amino acids were analysed by Alphalyse (Denmark) and performed on a Biochrom™ amino acid analyzer.

### Reporter Assay

The hSEAP-hFc reporter constructs containing the SEAP gene were analyzed using the QUANTI™-Blue™ SEAP Reporter System (Invivogen), which detects SEAP enzyme activity. Instead of a single reading after a determined period of time, multiple reads were taken at 1 min intervals for 20 minutes, after 30 minutes of incubation at 37°C, and used to express SEAP activity as absorbance units per second (abs/sec). The SEAP activity was normalized for integral cell area to generate relative specific productivity[19].

## Results and Discussion

### Growth Kinetics

**Figure 1:**
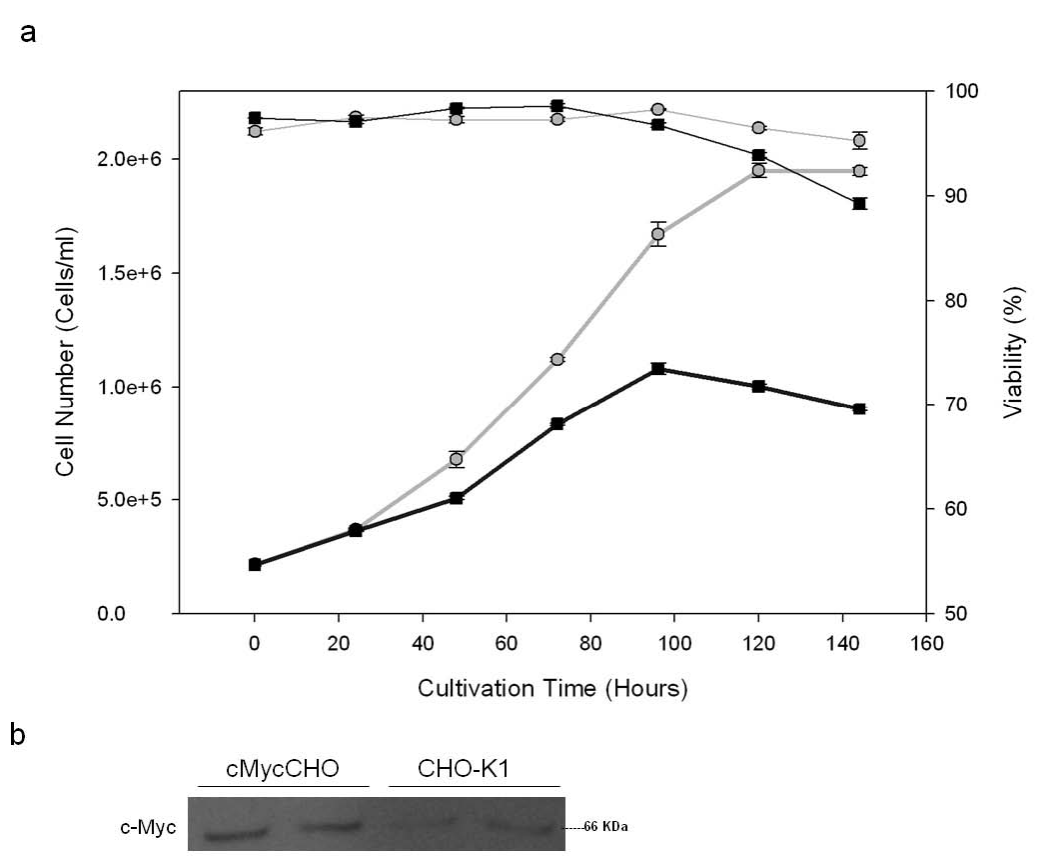
**Batch cultivation of cMycCHO**. The growth curve (a) of cMycCHO (grey circles) compared to CHO-K1 (Black squares). This culture seeded and grown (n = 3) in parallel, as a control for the BrdU pulse labeled cultures shows that cMycCHO (maximum cell density 1.99 × 10^6 ^cells/ml) has an increased proliferation capacity compared to the CHO-K1 (maximum cell density 1.1 × 10^6 ^cells/ml). (b) Western analysis of biological duplicate samples with c-Myc over-expression observed in the cMycCHO cell lines.

Figure 1a shows the growth curve of cMycCHO compared to CHO-K1. The adherent cMycCHO culture showed a significant increase in overall proliferative capacity being able to proliferate to a cell density of 1.99 × 10^6 ^cells/ml, a 72% increase in density versus the control CHO-K1. The viability remained above 90% for both cell lines throughout the culture until day 6 when the CHO-K1 viability fell just below 90% compared to 95% from the cMycCHO culture. The growth rate increased from 0.017 hours^-1^, for CHO-K1, which is comparable to reported rates of other adherent CHO cultures [3,20,21], to 0.023 hours^-1^, for cMycCHO. This resulted in a doubling time decrease of 25.4% while the integral viable cell (IVC) increased from 4.34 × 10^9 ^cell day L^-1 ^to 6.87 × 10^9 ^cell day L^-1 ^for the CHO-K1 and cMycCHO, respectively for the period of 5 days. From current and previous work it is seen that cMycCHO cells have a different morphology in adherent culture to CHO-K1. This is further investigated by measuring cell size. Contact inhibition may be a factor, as well, in determining cell number but in this case both cell lines allow for some cells to grow over neighbours. The increase in IVC would be an advantage in bioprocess systems where the increase in overall maximal cell number usually associated with more product formation if cell production rate is not significantly decreased.

The constitutive over-expression of *c-myc*, has clearly resulted in an overall increase in proliferation rate but also an increased maximal cell number under batch conditions. Increased proliferation would mean that cell division has increased or apoptosis has decreased or both. The fact that c-myc has been shown to be a potent inducer of apoptotic cell death in mammalian cells [11] in contrast to the viability data obtained in this work warrants further studies to explore the control mechanism of cell growth/death kinetics.

In addition, in order to confirm that the over-expression of the c-Myc protein was still present after thawing the cell line a western blot was done from duplicate cultures derived from the experimental seeding inoculum and grown to mid-exponential phase to obtain the supernatant for analysis shown in Figure 1b.

### Cell Cycle

**Figure 2:**
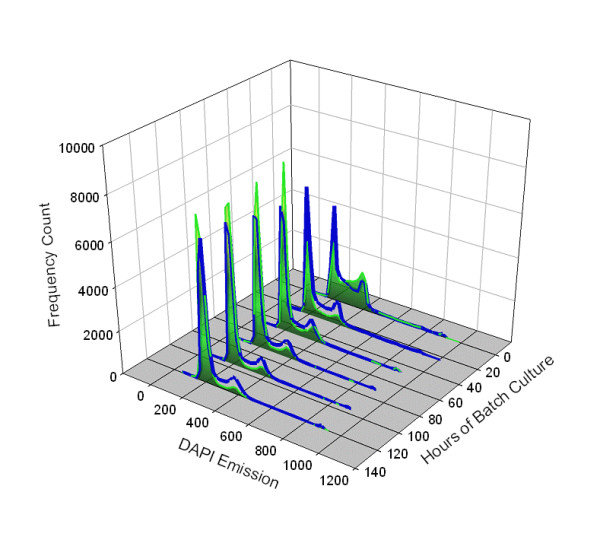
**A histogram of the cell cycle phases for cMycCHO versus CHO-K1 culture**. cMycCHO is represented by the non-shaded cell cycle profile (blue profile line) while the CHO-K1 profile represented by a shaded area underneath the histogram. It can be seen that the percentage frequency of the G_1_/G_0 _region in the 200 channel emission spectra decreases below CHO-K1 as the culture progresses while the S-phase (between 200 and 360 emission channel) and G_2_/M (at ~360 emission channel) is consistently greater with culture time for cMycCHO.

The cell cycle distribution of cMycCHO cells in Figure 2 clearly shows a consistent increased proportion of post G_1 _phases over the control (on average >10% increase in S phase cells). However, at the end of the batch cultures both cultures showed an increase in G_1 _cells indicating a decline in proliferation rate presumably due to increasing environmental stress resulting from nutrient deprivation and accumulation of toxic metabolites. Previously, it was shown in CHO cultures that the proportion of S phase cells enables prediction of changes in cell number and reflects the proliferation dynamics of cell culture [22]. It may be reasonable to suggest that the increased growth rate of cMycCHO is a consequence of increased proportion of S phase cells.

**Figure 3:**
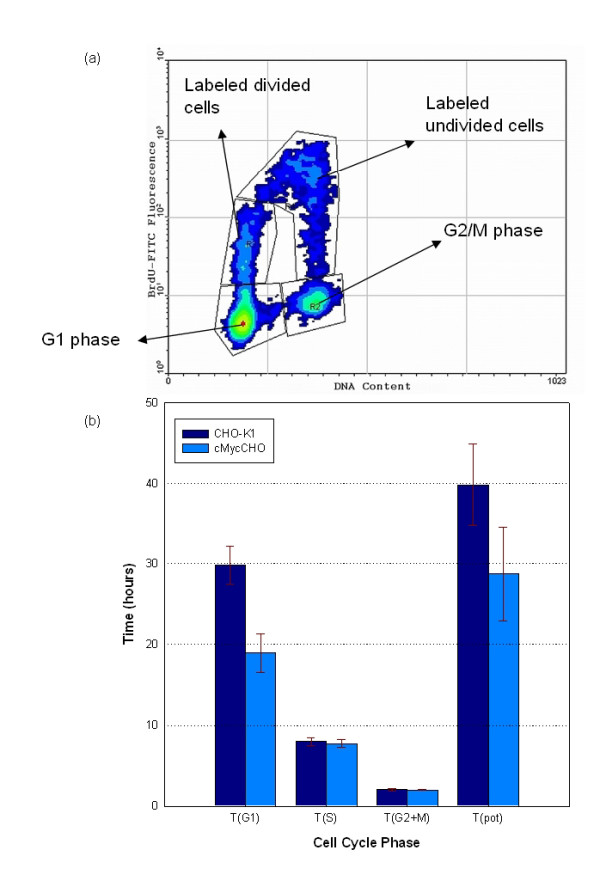
**Cell cycle kinetics analysis**. Example of the contour plot obtained 5 hours after BrdU pulse labelling cMycCHO culture (a). The plot displays the DNA content (x-axis) and BrdU-FITC fluorescence intensity (y-axis). Four regions can be observed with the G_1 _and G_2_/M phase represented by the DNA content shift while the BrdU labeled undivided cells undergo a temporal shift from the region above G_2_/M to the region above G_1_, indicated as the labeled divided cells on the plot. The bar chart (b) represents the cell cycle phase times: TG1, TS, TG2+M, and Tpot (n = 3). Unpaired Student's t-test indicates TG1 and Tpot are significantly different between cMycCHO and CHOK1 (p < 0.05).

Figure 3a is a representative bi-variate contour plot at 5 hours after pulse labelling during exponential phase illustrating cells in G_1 _and G_2_/M phase as well as the undivided (f_lu_) and divided (f_ld_) fractions. From the plots the relative movement of labeled undivided cells as a function of time was calculated and a linear regression analysis was applied to determine the cell cycle times (Figure 3b). The values for T_S _were similar for both cultures but when the potential cell cycle time (T_pot_) was calculated, cMycCHO showed a significantly faster time. Subsequently the time spent in G_1 _phase was found to decline by 36.4% in cMycCHO which pertains to the increased proliferation rate observed. It was obvious that the increase in proliferation rate of cMycCHO was due to a decreased cell cycle time via a shortened G_1 _phase. The finding of this work is in agreement with previous work showing that deletion of the c-myc gene impedes cell cycle progression thus retarding cell proliferation [23,24]. More importantly, the analysis gives a clear indication that *c-myc *over-expression enhances cell proliferation by promoting the earlier G_1_-S transition, by shortening the G_1 _phase of the cell cycle. It appears that once the cell progresses to S phase of the cell cycle the time to cell division remains unchanged.

### Cell Size

**Figure 4:**
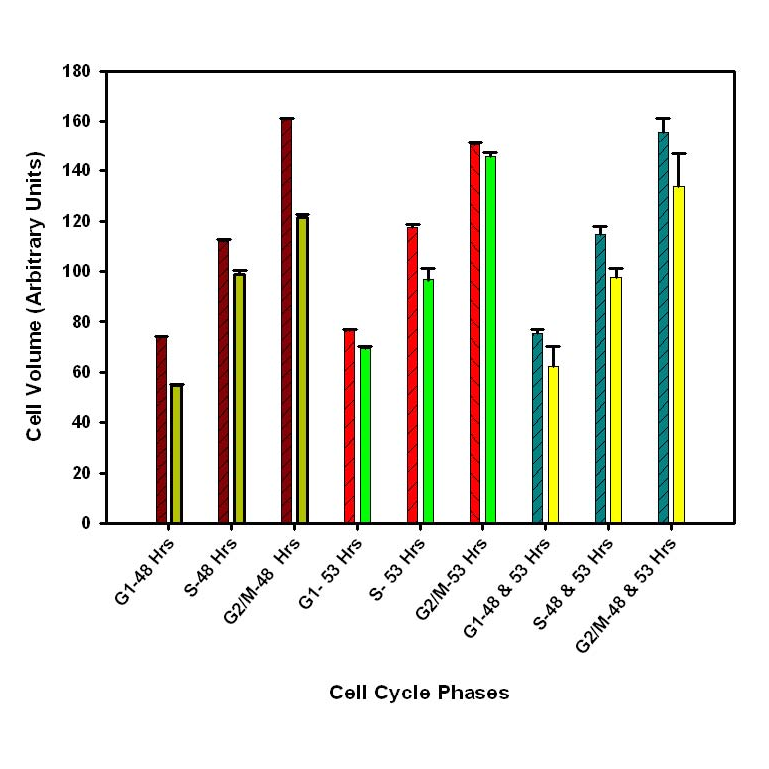
**Cell size at different cell cycle phases for cMycCHO (right bar) versus CHO-K1 (left bar) at 48 and 53 hours of the exponential phase culture with n = 3**. The third set is a combination of both samples to illustrate the smaller cell size of cMycCHO from G1 and carried throughout the cycle.

**Figure 5:**
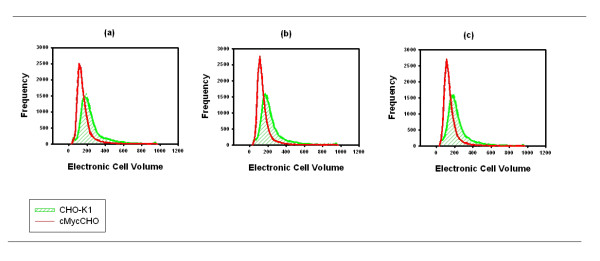
**Electronic cell volume distributions (arbitrary units) of cMycCHO (red line) and CHO-K1 (green, shaded) cell lines (n = 3) from early-24 hours (a), mid-72 hours (b) to late-96 hours(c) exponential phase**.

Throughout the exponential phase of the cell culture the cell size of cMycCHO is shown to be smaller than the control. Quantifying the relative cell size difference for each phase of the cell cycle it is shown that the exponential growth occurs with cMycCHO having a smaller size throughout (Figure 4). With an average cell diameter of 12.16 μm and 14.11 μm respectively (Figure 5), While this represents a 36% decrease in cell volume, it indicates a difference in the mean volumetric biomass (a function of the cell volume and maximum cell density) of 1:0.9 nm^3 ^cells ml^-1 ^for cMycCHO:CHO-K1, a rather minor difference in comparison to the >70% difference in cell number.

Beier et al. (2000) suggested that *c-myc *over-expression may control cell size [25]. However, Trumpp et al. (2001) argued that *c-myc *controls cell number and not the cell size. Indeed, fibroblasts have shown no need for *c-myc *activity for increasing cell size but such activity was necessary for G1-S transition [26]. Other studies have reported varied results for the relationship between *c-myc *and cell size. For example, hepatocytes with *c-myc *overexpression have shown an increased cell size in one study [27] and reduced cell size in another study [28]. An increase in cell size may well depend on the stoichiometric molar concentrations of *c-myc *leading to the cell size increase through the simulation of mTOR via inhibition of TSC2 [29], which might be the case in some cancers. While CHO cells have many similarities to cancerous cells the contrasting results of decreasing cell size may not find explanation in mTOR pathway and protein synthesis. However, it is not unreasonable to suggest that such a significant change in size would involve a major change in the metabolic consumption and secretion state of the cells, since the cell's ability to either get substances from the outside, or eliminate waste, is related to the surface area. Since the increased cell number of cMycCHO was independent of nutrient availability the concentration of necessary nutrients and energy required for maintenance and proliferation should be reduced if apoptosis is not enhanced; a suggestion is investigated below. It may be interesting to note that when a cell enters the cell cycle it has to reach a critical mass before proceeding to S phase. This work shows that *c-myc *can cause a reduction in the required critical mass for the transition into S phase by decreasing protein content (data not included) in addition to cell size. This suggests a decoupling of cell size from cell division, a phenomenon that has been also observed in stem cells and neurons, and in addition, elevated G_1 _cyclin levels in fibroblast cells can cause elimination of restriction point control resulting in smaller small cell size [30,31].

### Metabolites

Table 1 shows the specific consumption and production rates of several metabolites. Glucose analysis revealed that cMycCHO had a 40% decrease in specific glucose consumption rate. This decreased value is associated with the decrease in specific lactate production rate (43%). Glutamine consumption was also decreased by 42% while ammonia production showed only a slight increase of 9%. Lactate/glucose molar yield coefficient has remained relatively similar in both cell lines, but the yield coefficient for ammonia from glutamine has increased by 47% mainly due to the decrease in glutamine consumption rate. The increased ammonia production is consonant with the view that alanine is metabolised to form glutamate, which is subsequently deaminated oxidatively to liberate ammonia. The association of such an increase with *c-myc *is not clear but lower rate of glutamine consumption is consistent with lower maintenance energy requirement by cells at higher growth rates.

**Table 1:** Specific rates for glucose, lactate, l-glutamine, and ammonia for cMycCHO and CHO-K1 cell lines.

**Parameters for CHO Cell Culture**		
	**CHO-K1**	**cMycCHO**

q_Glc_(μmol 10^-6 ^cell day^-1^)*	6.25	3.74
q_Lac_(μmol 10^-6 ^cell day^-1^)	6.50	3.72
q_Gln_(μmol 10^-6 ^cell day^-1^)*	0.88	0.51
q_Amm_(μmol 10^-6 ^cell day^-1^)	0.59	0.65
*Y *'_Lac/Glc_(mol/mol)	1.04	0.99
*Y *'_Amm/Gln_(mol/mol)	0.67	1.27

* Consumed metabolite

**Figure 6:**
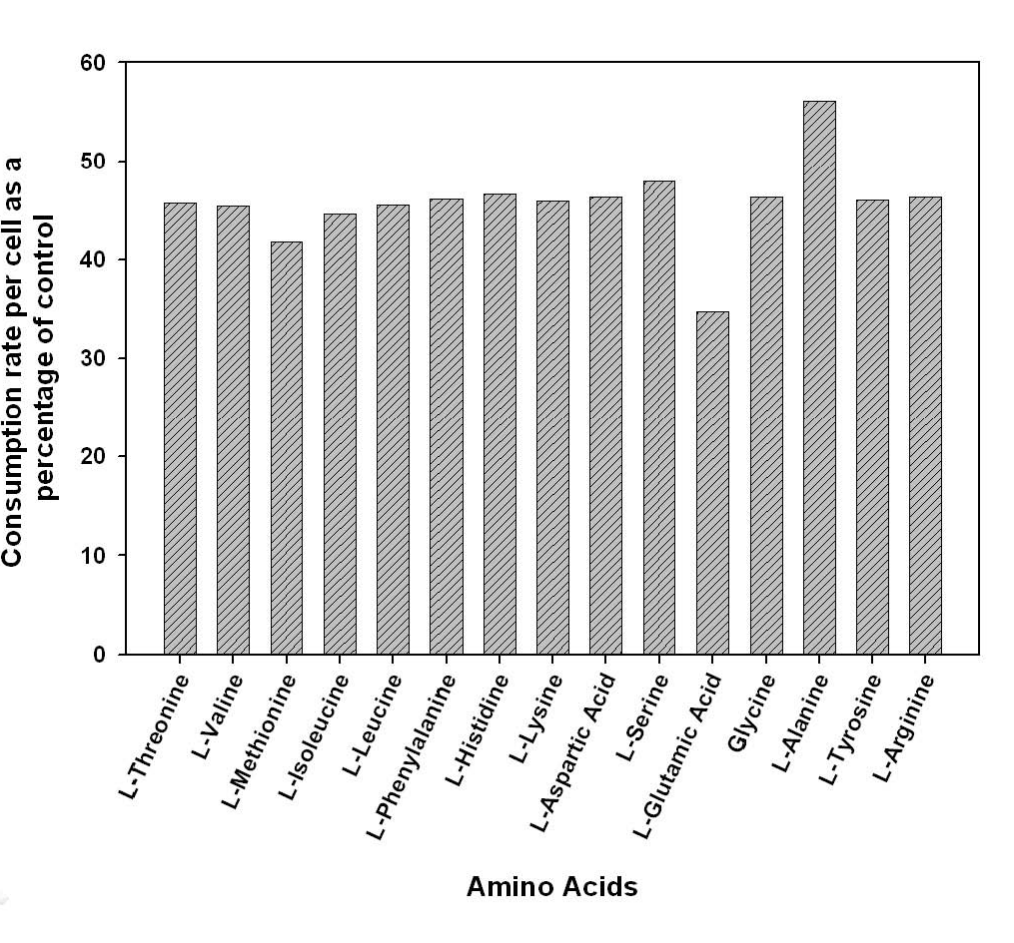
**Amino acid consumption in cMycCHO cell line as a percent of the control cell line illustrated by the ratio of amino acids consumed per cell at the end of the exponential growth phase (day 5)**.

Individual amino acid consumption rates, shown in Figure 6, display an overall reduction in amino acid uptake at the end of exponential phase of cMycCHO culture. The highest decrease in amino acid consumption rate is seen for glutamate, with 65% decrease in consumption. The difference in total amino acid consumption rate is approximately 2.2 fold apart from glutamate (2.8 fold) and alanine (1.8 fold). The lower fold change for alanine and increased fold change in glutamate may be related to a difference in alanine transaminase reaction between the two cell lines, where cMycCHO has an increase in the amount of nitrogen being incorporated into alanine [32]. The by-product of this reaction is pyruvate which can be used in glycolysis to lower glucose consumption and lactate production [33,34]; an explanation supported by the current metabolic data (Table 1). Moreover, the lowered metabolic burden of a smaller cell size may also offer an explanation to the reduced metabolic requirements of the cMycCHO cells. It may also possible that the decreased cell volume could mean that the energy requirements to sustain other protein synthetic machinery, not essential for cell survival, have also decreased.

Additionally, since the mTOR pathway and c-Myc protein are affected upstream by AKT [35,36], over-expression of c-Myc protein will bypass this pathway and partly decouple the simultaneous effect on both growth and proliferation. Thus, since c-Myc initiates entrance into S-phase, by acting as a transcription factor for several cell cycle genes such as cyclin D2 and CDK2 [9], and since the mTOR pathway suggests the same AKT activation from the external environment as the control, cell size would not increase with increased proliferation thus decreasing the overall cell size distribution.

These studies together with the current data suggest that the decreased influx of amino acid is a consequence of the decreased critical mass required to enter S-phase. In limited cellular resources c-myc forces the cell to progress prematurely into S phase.

### Apoptosis

**Figure 7:**
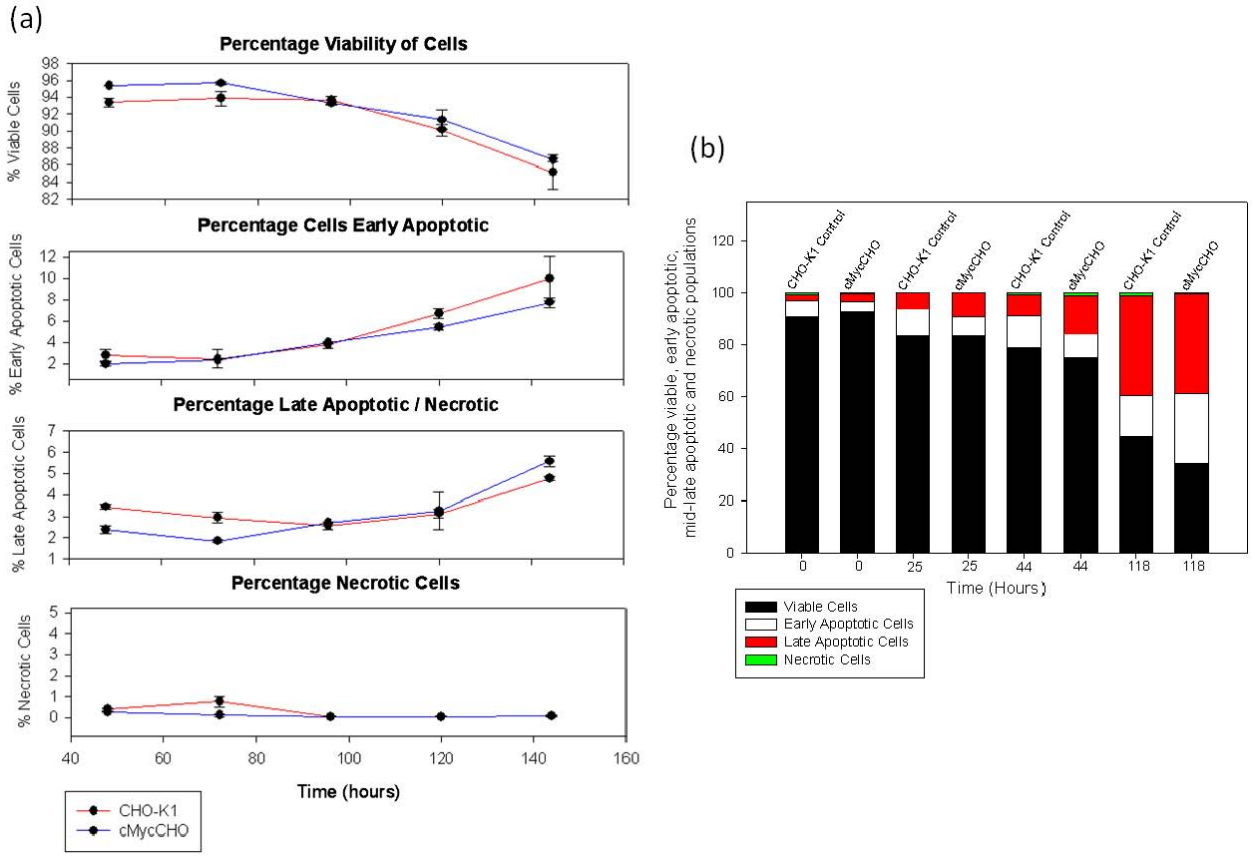
**Apoptosis of cMycCHO compared to CHO-K1 cultures in exponential growth (a) and spent medium (b)**. Cells were analysed by staining with Annexin V-FITC and PI for counting viable (none stained), early apoptotic (Annexin V-FITC staining), late apoptotic (both Annexin V-FITC and PI staining) and necrotic cells (PI staining only) at 0, 25, 44, and 118 hours. Under t-Test criteria, p < 0.05 for viable and apoptotic cells with n = 3. Spent medium was obtained from the end of the death phase of batch culture. Cells from both cell lines were incubated in spent medium for up to 118 hrs.

The data in Figure 7 demonstrates that apoptosis plays a minimal part in determining the cell number during the growth phase. Contrary to previous results the cMycCHO cell line showed a slight decrease in apoptosis during the growth and decline phase of cell culture in comparison to the control. The data demonstrated that *c-myc *did not sensitised the cells to apoptosis stressors. The data are consistent with the control and *c-myc *over-expressed cells being equally killed by apoptosis at the later stage of cultures. Quantification of apoptosis in spent media cultures (Figure 7b) gave similar results to the batch culture where a minimal difference was observed between the two cell lines. After 118 hours in spent media both cell lines had 38% of late apoptotic cells in culture. Thus, it is most unlikely that *c-myc *plays any role in CHO apoptosis which triggers in cell culture by nutrient depletion and environmental chemical stresses [37-40].

### Cell Growth and hSEAP-hFc Production

**Figure 8:**
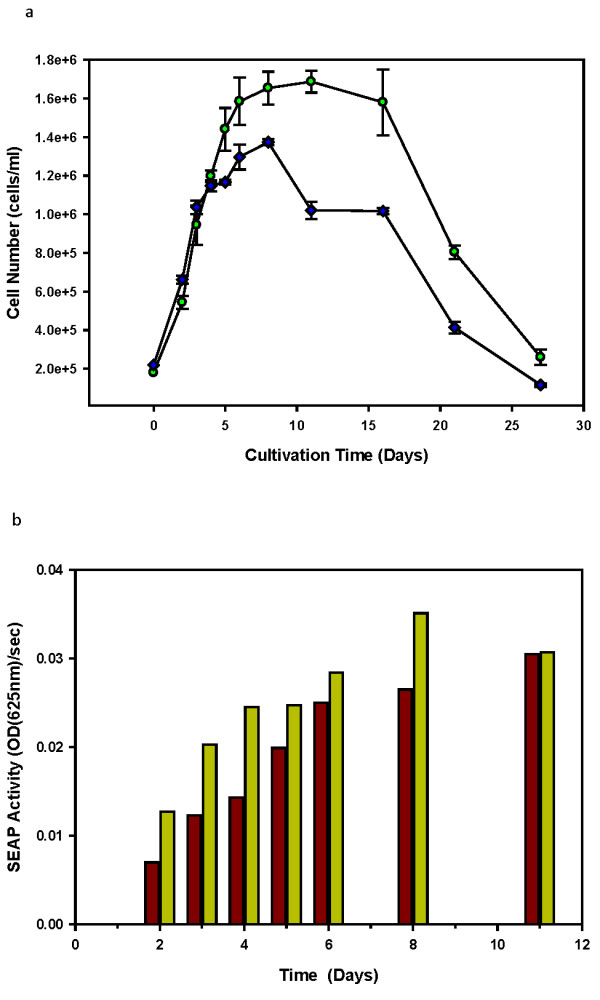
**Batch Cultivation of CHO95 versus CHO80**. This is represented by the growth curve (a) of CHO80 (blue diamonds) and CHO95 (green circle) cell line (n = 3) including the (b) SEAP activity of CHO80 (yellow bar) versus CHO95 (brown bar) measured from the supernatant by kinetic analysis of 625 nm absorbance through 60 second intervals for 50 minutes for time points representing the culture progression from exponential phase to the start of the death phase.

To understand if a transfected and selected stable population of cMycCHO cells can affect the general production of a fusion SEAP compared to the CHO-K1 population, the cells were analysed for production capability. Upon transfection with a hSEAP-hFc construct and selection of the cMycCHO cell line population, the procedure produced a cell line with an increased maximal cell density (CHO95, using the *c-myc *over expressing cell line) compared to the control CHO80 as can be seen from the resultant growth curves (Figure 8a). The transfected CHO-K1-hSEAP-hFc (CHO80) remained at a lower maximal cell number of 1.37 × 10^6 ^cells/ml compared to the 1.69 × 10^6 ^cells/ml reached by CHO95. The growth rate between the cell lines has a difference of 4.4 hours in the doubling times with CHO95 obtaining the fastest doubling time of 34 hours with high confidence of p < 0.05 using the Student's *t*-Test. This result shows that in the two stably selected cells, the *c-myc *gene bestows greater proliferation even with the extra metabolic burden of recombinant protein production.

The hSEAP-Fc activity was measured to be greatest throughout the culture supernatant for CHO80 (See Figure 8b), thus the secretion of properly functioning hSEAP-hFc was higher in this cell line. The assay used the kinetics of hSEAP-hFc to determine activity rate via linear regression analysis obtaining an indirect measurement of productivity. As can be seen only on day 11 of the culture, when CHO80 has entered the death phase, and CHO95 is in stationary phase, the amount of hSEAP-hFc activity for each population is within 0.7% of each other while the relative specific productivity of CHO95 is 87.38% of CHO80. This indicates that the CHO95 cell line with *c-myc *over-expression has lower hSEAP-hFc productivity. There appears to be a consequence to the increased growth profile of *c-myc *expressing CHO cells which is a reduction in the rate of active hSEAP-hFc production.

## Conclusion

While over-expression of c-Myc reduces the transition time of G_1 _phase to S, the resultant reduction in the critical mass required for this earlier transition to occur negatively affects the maximal volumetric biomass achieved in culture. In addition, the overall metabolic energy pathways are modified to decrease glucose and glutamine consumption rate which might explain the resultant increase in maximal cell density in the absence of nutrient supplementation. The shortening of the G_1 _phase by *c-myc *over-expression allowed the cells to have higher growth rate while reducing cell size allowing cells to achieve higher cell numbers. Apoptosis was shown to be unaffected by *c-myc*, in contrast to previous reported results but production of hSEAP-hFc from a constitutive *c-myc *over-expressing cell line did not increase with the increase in cell number.

This report clearly demonstrated that by manipulation of the cell cycle kinetics, and indirectly its metabolic pathways, it is possible to reach higher cell densities in batch cultures with limited nutrients. However, the effect on recombinant protein production due to the smaller cell size and reduced metabolite consumption will need to be further investigated to establish *c-myc *usefulness in biologics production.

## Authors' contributions

DK and MAR designed the research. DK performed all the experiments and analyzed the data. MAR conceived the study, and participated in its coordination. DK wrote the manuscript. Both authors read and approved the final manuscript.
